# Upcycled PET‐Derived Carbon Foam Functionalized with Cu_3_SbS_4_–Sb_2_S_3_ Heterostructures for Efficient Interfacial Solar Desalination

**DOI:** 10.1002/smll.202506862

**Published:** 2025-08-20

**Authors:** Muzammil Hussain, Kassa Belay Ibrahim, Enrique Rodríguez‐Castellón, Silvia Gross, Pawan Kumar, Stéphanie Bruyère, David Horwat, Elisa Moretti, Alberto Vomiero, Tofik Ahmed Shifa

**Affiliations:** ^1^ Department of Molecular Sciences and Nanosystems Ca' Foscari University of Venice Via Torino 155 Venice 30172 Italy; ^2^ Department of Industrial Engineering University of Padova Via Venezia 1 Padova 35131 Italy; ^3^ Department of Inorganic Chemistry Crystallography and Mineralogy Faculty of Science Inter‐university Institute of Research in Biorefinery I3B University of Malaga Málaga Spain; ^4^ Department of Chemical Sciences University of Padova Via Francesco Marzolo 1 Padova 35131 Italy; ^5^ Division of Materials Science Department of Engineering Sciences and Mathematics Luleå University of Technology Luleå 97187 Sweden; ^6^ Université de Lorraine CNRS IJL Nancy F‐54000 France

**Keywords:** chalcogenides, circular economy, photothermal evaporators, plastic upcycling, solar desalination

## Abstract

Solar desalination is an emerging technique to produce fresh water utilizing renewable solar energy. However, the engineering of efficient photothermal material is a significant obstacle. In the present study, a carbon foam is synthesized from the upcycling of waste PET and hydrothermally functionalized with a heterostructure composed of Cu_3_SbS_4_ and Sb_2_S_3_. Material characterizations demonstrated the successful decoration of nanochannels on graphitic carbon foam (CF). The analysis of the optical properties in the UV/Vis‐NIR spectral range demonstrated excellent absorption properties of 96% for Cu_3_SbS_4_‐Sb_2_S_3_/CF compared to Sb_2_S_3_/CF (48%) and CF (68%) in near‐IR. Photothermal desalination results reveal the evaporation rate of 2.82 kg m^−2^ h^−1^ for Cu_3_SbS_4_‐Sb_2_S_3_/CF compared to  1.4 kg m^−2^ h^−1^ for Sb_2_S_3_/CF and  1.58 kg m^−2^ h^−1^  for CF, with 99% salt removal in condensed water. The formation of the composite leads to a high surface temperature and enhanced evaporation rate. The contact angle analysis confirmed the hydrophilic nature of the material that plays a crucial role in the solar desalination process. These findings elucidate the effective photothermal performance achieved through chalcogenide heterostructure engineering supported on upcycled carbon foam derived from waste PET, demonstrating a practical application aligned with circular economy principles in solar desalination.

## Graphical abstract

Carbon foam is upcycled from waste PET and decorated hydrothermally with Cu_3_SbS_4_‐Sb_2_S_3_ to prepare a photothermal evaporator. The Cu_3_SbS_4_‐Sb_2_S_3_/CF reveals superior photothermal performance with excellent evaporation rate under one‐sun radiation. This renewable‐based sustainable strategy enables circular economy practices that suggest a promising solution to mimic water scarcity through solar steam generation.

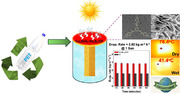

## 1 Introduction

Fresh and clean water is a basic need of humans and is highly associated with human survival. However, due to the blooming in industrialization and urban development, the water resources are highly affected.^[^ ^1^, ^2^, ^3^ ^]^ The utilization of seawater for drinking and energy production has consistently raised concerns, prompting the development of various strategies for desalinating seawater. A series of energy‐intensive methods with complex technologies are used for water desalination, including electrodialysis (ED), forward osmosis (FO), multiple‐effect distillation, reverse osmosis (RO), thermal desalination, solar desalination, and multi‐stage flash distillation.^[^ ^4^ ^]^ Among them, solar desalination is one of the emerging methods to treat seawater using renewable solar energy. Interfacial solar steam generation using solar energy is not only a cost‐effective and straightforward technology, but it also harnesses a free and clean energy source.^[^ ^5^ ^]^ In photothermal material for desalination, the solar energy can be transformed into thermal energy that increases the water evaporation rate and makes the process more efficient and greener under sunlight irradiation.^[^ ^4^, ^6^ ^]^

Material development for solar steam generators plays a significant role in enhancing the solar absorption and evaporation rate. Considering these concerns, in recent years various carbon materials have been investigated, such as graphite, graphene oxide, CNTs, biomass, and wood‐derived carbonized carbon.^[^ ^7^, ^8^, ^9^, ^10^, ^11^ ^]^ These carbon‐based materials have similar properties, such as good light absorption and lightweight.^[^ ^5^ ^]^ On the other side, carbon‐based systems are missing some key features that are required for high solar desalination, they may have high synthesis cost (graphene oxide and biochar), low water transport and light reflection due to the layered structure (graphene oxide), and hydrophobic nature (CNTs /graphene), which reduces the water evaporation rate.^[^ ^12^, ^13^ ^]^ Furthermore, the high thermal conductivity of these carbon materials reduces the surface thermal properties, favoring heat dispersion in the water reservoir, reducing an efficient interfacial solar desalination.^[^ ^14^ ^]^ The development of cost‐effective carbon materials with low thermal conductivity, hydrophilicity, and high solar absorption, such as PET (polyethylene terephthalate) upcycled carbon foam, could be an appealing solution. Waste PET upcycled carbon foam is an excellent carbon material owing to the essential characteristics of solar evaporators, such as high hydrophilicity and low thermal conductivity, which prevent the surface heat loss and boost the evaporation rate.^[^ ^15^ ^]^

Semiconductors, plasmonic and hydrated transition metal chalcogenides, based nano‐evaporators play a crucial role in the solar steam generation.^[^ ^16^, ^17^, ^18^, ^19^, ^20^ ^]^ A bibliometric analysis shows that in recent years the nanoevaporators have attracted significant interest from the research community, as evidenced by a substantial increase in related publications.^[^ ^6^ ^]^ Efficient solar steam generator design requires high solar absorption from UV/Vis to the Near Infrared region to guarantee absorbance of the full Sun spectrum, and a series of advanced materials is intensively investigated to obtain solar evaporation rates above 3 kg m^−2^ h^−1^.^[^ ^21^ ^]^ In this context, sulfide‐based materials attract strong interest in harvesting solar energy to produce thermal energy. Recently, copper‐based sulfide nanostructures have proved their excellent potential as solar steam generators.^[^ ^22^, ^23^, ^24^, ^25^, ^26^ ^]^ Sulfide‐based nanostructures are characterized by their narrow band gaps (usually ranging from 1.0 to 2.4 eV), and this electronic property permits them to absorb a broad spectrum (UV/Vis‐NIR) of solar radiation, which underlines the potential of sulfide‐based nanostructures in advancing efficient and sustainable solar desalination processes.^[^ ^27^ ^]^ Recently, ternary chalcogenides of copper‐antimony‐sulfide have attracted significant attention for solar cell applications due to their earth‐abundancy and low toxicity.^[^ ^28^, ^29^, ^30^ ^]^ They have demonstrated promising achievements due to their high optical activity, catalytic surface properties, and tunable morphologies, with encouraging performances in areas such as dye degradation and CO_2_ reduction.^[^ ^31^, ^32^, ^33^ ^]^ However, despite their intriguing optical and thermal characteristics, their potential as photothermal materials for interfacial solar desalination remains largely unexplored. Interfacial solar desalination demands a highly efficient combination of materials that, at least, meet three essential criteria: high hydrophilicity coupled with low thermal conductivity, suitable wettability, and excellent solar light absorption^[^ ^34^ ^]^. Herein, we report a rationally designed heterostructure composed of Cu_3_SbS_4_ and Sb_2_S_3_ (Stibnite) on carbon foam (CF). The CF is derived from the upcycling of waste PET. The engineered copper–antimony sulfide‐based heterostructure grown onto CF offers several key advantages for solar water desalination, including enhanced light absorption across a broad spectrum, improved photothermal conversion efficiency, and effective interfacial heat localization (due to the low thermal conductivity, porous and hydrophilic nature of the material which guarantee effective interfacial heat exchange between the evaporator and the absorbed water, resulting in a high evaporation rate). These features collectively make it an excellent candidate for achieving a high water evaporation rate under solar illumination. The obtained results prove that the heterojunction of Cu_3_SbS_4_ and Sb_2_S_3_ with CF successfully engineers the morphology of the hybrid material and enhances the surface thermal property as compared to Sb_2_S_3_/CF and the bare CF. In general, the combination of waste plastic‐derived graphitic carbon with sulfide material for the rational design of an efficient solar steam generator promotes circular economy practices while enabling clean water production.

## 2 Results and Discussion

**Figure 1:**
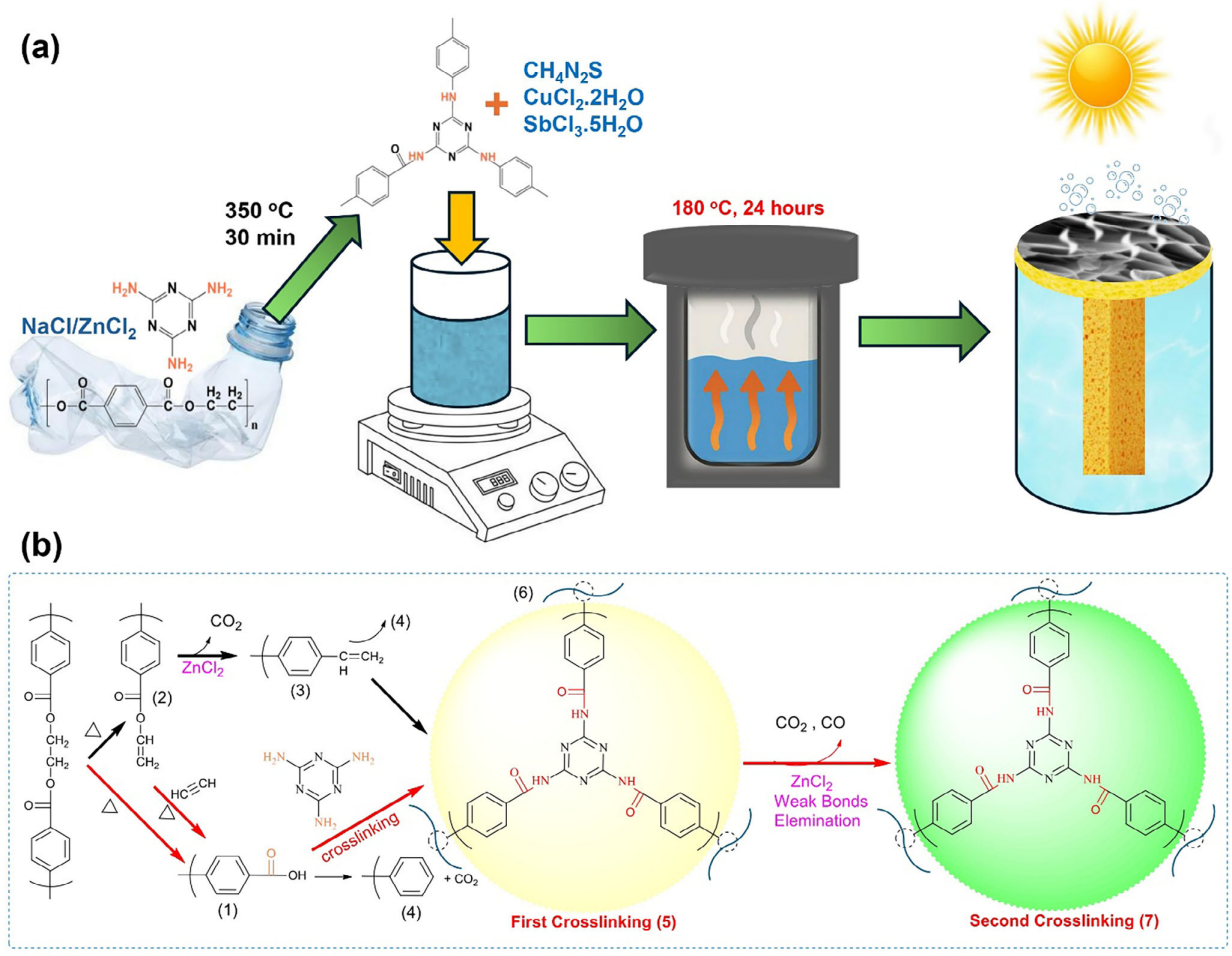
a) Graphic presentation of CF production and decoration of Cu_1_Sb_1.5_S on CF for interfacial solar desalination, b) Carbonization mechanism for CF formation.

**Figure 2:**
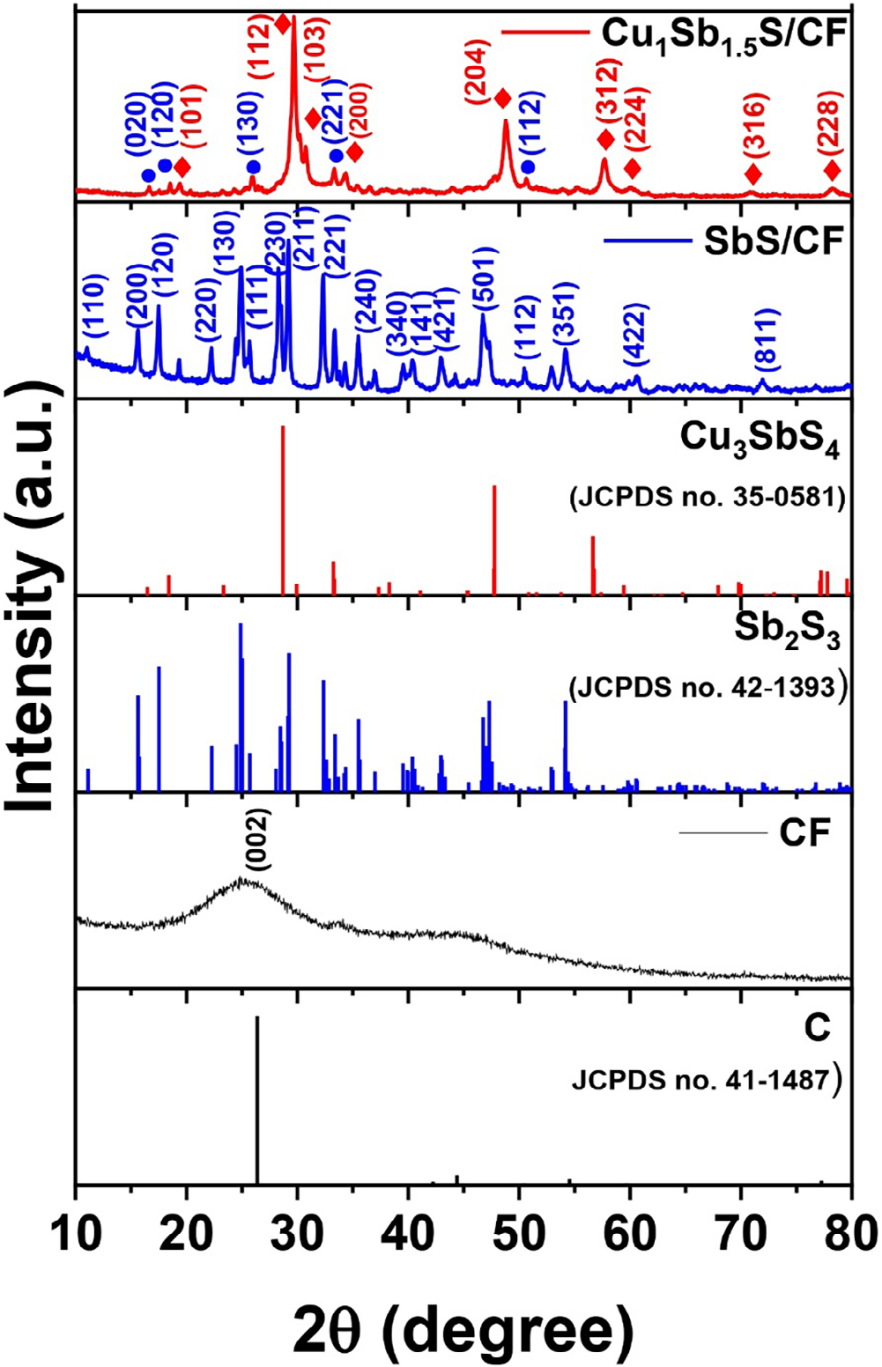
X‐ray diffraction patterns of CF, SbS/CF, and Cu_1_Sb_1.5_S (a), and Cu_1_Sb_1.5_S /CF and JCPDS cards for C, Sb_2_S_3,_ and Cu_3_SbS_4_ are also reported for direct comparison with the experiments.

Initially, the carbon foam (yield = 35.95%) was synthesized from PET of waste plastic bottles using eutectic salt of NaCl and ZnCl_2_ and melamine by carbonization in an air environment. To synthesize an efficient photothermal layer, the carbon foam was further functionalized hydrothermally by copper–antimony sulfide‐based materials and investigated for interfacial solar desalination, see **Figure** **1**a. The conversion mechanism of PET to carbon foam is illustrated in Figure 1b, which starts with the degradation of the PET chain by random scission at the ester linkage (beta‐hydrogen transfer), which creates two chain fragments (1‐carboxyl‐terminated chain and 2‐vinyl‐terminated chain). The vinyl‐terminated fragment (2) decomposes into a carboxyl‐terminated fragment (1) by eliminating acetylene. During the first crosslinking, the amide reaction of the carboxyl group of the carboxyl‐terminated chain and the amino group of melamine causes carboxyl‐terminated PET degradation intermediate (5). The crosslinking of melamine with a carboxylic group suppresses the formation of aromatic ring‐terminated fragments (4) from the carboxyl‐terminated chain (2) and (3), and during the carbonization process, the decarboxylation and dehydration result in the formation of a stable framework due to the catalytic action of molten salt by owing to the Lewis acidity of ZnCl_2_. The compound (3) from vinyl‐terminated chain fragments undergoes intermolecular condensation to ease the self‐crosslinking structure (6). Zinc chloride not only promotes the decarboxylation but also removes the weak bonds of the crosslinked structure, which yields a more stable crosslinking structure (7). During the degradation and crosslinking process, the physical template effect of NaCl and in situ formed small molecular compounds such as CO_2_ and H_2_O causes a foaming effect. X‐ray diffraction analyses were performed to investigate the crystal structure of the synthesized materials (namely: CF, SbS/CF, and Cu_1_Sb_1.5_S/CF) in the angular range of 10°‐80°. **Figure** **2** shows the broad Bragg peak at 2θ = 18°–32°, attributed to the (002) diffraction by graphitic carbon of CF (JACPDS no. 41‐1487). The Sb_2_S_3_ grown on CF exhibits diffraction peaks at 2θ values of 11.1, 15.7, 17.5, 22.2, 24.8, 25.7, 28.4, 29.2, 35.5, 39.9, 40.3, 42.9, 46.7, 48.7, 52.9, 60.6 and 71.9° the crystal planes (110), (200), (120), (220), (130), (111), (230), (211), (221), (340), (141), (421), (501), (112), (351), (422), and (811) of stibnite (JACPDS no. 42‐1393). The addition of Cu precursor during the hydrothermal synthesis induces the advent of another phase in addition to stibnite. The Cu/Sb ratio of 1:1.5 mm resulted in a heterostructure in which two different phases, stibnite (Sb_2_S_3_) and famatinite (Cu_3_SbS_4_) co‐exist. As illustrated in Figure 2, the Cu_1_Sb_1.5_S/CF sample exhibits the diffraction peaks at 2θ values of 18.4, 28.6, 29.9, 33.2, 47.7, 56.6, 59.4, 77.2 and 89.1° that can be indexed to Cu_3_SbS_4_ (JACPDS no. 35‐0581), with crystal planes of (101), (112), (103), (200), (204), (312), (224), (316), and (228), respectively. It is also evident from the Figure 2 that the peaks at 2θ value of 15.6, 17.5, 24.8, 32.3 and 48.7° can be indexed to Sb_2_S_3_ (JACPDS no. 42‐1393) with crystal plans of (020), (120), (130), (221), and (112) respectively. Moreover, the XRD patterns of samples with varied concentrations of Cu and Sb are presented in Figure S1a (Supporting Information). It is noteworthy that the CF did not induce any major crystal changes on the growth of the materials. This can be evidenced from Figure S1b (Supporting Information) where there is no significant alteration in the XRD pattern of Cu_1_Sb_1.5_S as compared to that of Cu_1_Sb_1.5_S/CF in Figure 2. Furthermore, the Rietveld Refinement was performed to determine the phase contents of the Cu_3_SbS_4_–Sb_2_S_3_ heterostructure. The refinement was performed using (X'pert High Score Plus), and structural models were based on standard crystallographic data (Cu_3_SbS_4_: JACPDS no. 35‐0581 and Sb_2_S_3_: JACPDS no. 42‐1393). The refined pattern exhibited good agreement with the experimental data, with a χ^2^ value of 5.28, indicating a reliable fit. Quantitative phase analysis revealed that the sample consists of ≈58 wt.% Cu_3_SbS_4_ and 42 wt.% Sb_2_S_3_ Figure S1c (Supporting Information). These results prove the structural integrity of the biphasic heterostructure (Cu_3_SbS_4_–Sb_2_S_3_).

X‐ray Photoelectron Spectroscopy (XPS) analyses were conducted to check the elemental composition and chemical state of CF and nanostructure functionalized CF. The XPS survey spectrum in Figure S2a (Supporting Information) shows surface chemical composition (in atomic concentration%) was: C: 74.0%, N: 13.0%, O: 5.6% and Cl: 1.1%. The high resolution C 1*s* core level spectrum of CF (Figure S2b, Supporting Information) shows four contributions at 284.8 eV (69%), 286.1 eV (18%), 287.2 eV (7%) and 288.6 eV (7%), assigned to adventitious carbon/─C─C─/─C═C─, C─OH/─C═N, C═O, and ─COOH, respectively. The high‐resolution O 1*s* core level spectrum (Figure S2c, Supporting Information) shows three contributions at 530.8 eV (10%), 532.0 eV (51%), and 533.4 eV (39%), assigned to C═O/─COOH and C─OH, respectively. The N 1*s* spectrum has two contributions with similar relative intensities at 398.6 and 400.2 eV, assigned to pyridinic N and graphitic N. In addition, this sample presents a small amount of chlorine at the surface with a Cl 2*p*_3/2_ signal at 197.3 eV, typical of chloride from final washing with HCl. Figure S3a (Supporting Information) presents the surface chemical composition of sample SbS/CF Figure S3b (Supporting Information) shows the C 1*s* core level spectra of the sample SbS/CF shows three contributions at 284.76, 286.2, and 288 eV assigned to /─C─C─/─C═C─, C─OH/─C═N, and ─COOH/─COO^−^. The N 1*s* in Figure S3c (Supporting Information) present similar information as observed in CF, with two deconvoluted peaks at 398.6 and 400.2 eV corresponding to pyridinic and graphitic carbon. The O 1*s* core level shows overlapping with Sb 3d_5/2,_ which presents two contributions at 529.9 and 539.2 eV corresponding to Sb 3d_5/2_ and the peak at 531.7 eV for C═O/─COOH (Figure S3d, Supporting Information). Figure S3e (Supporting Information) shows the deconvoluted peaks for the S 2*p* core level spectrum that presents two doublets S 2*p*_3/2_‐S 2*p*_1/2,_ with S 2*p*_3/2_ binding energy values at 162.6 and 168.01 eV corresponding to sulfide and sulfate.

**Figure 3:**
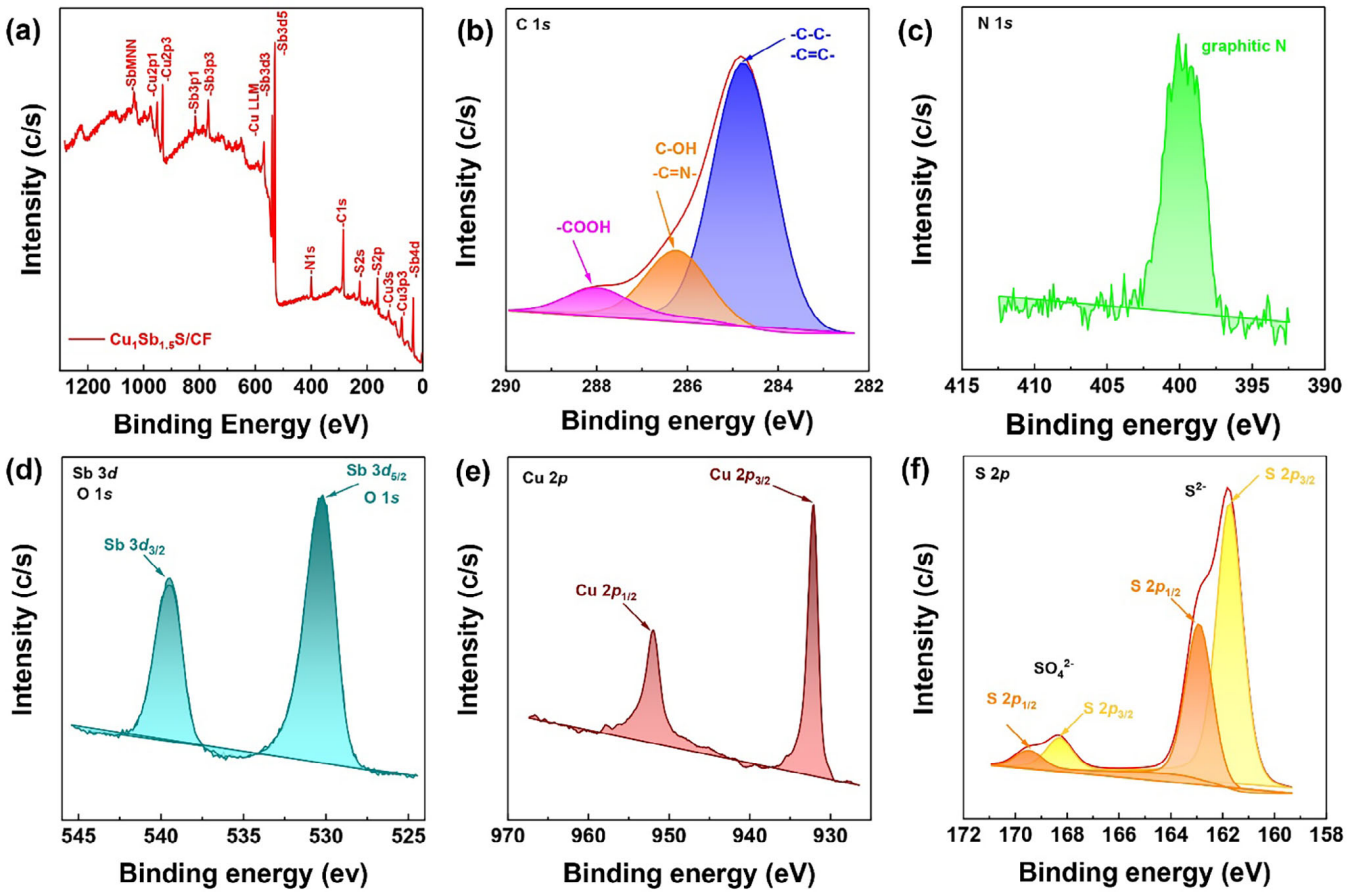
a) XPS survey spectrum of Cu_1_Sb_1.5_S/CF, high‐resolution b) C 1*s*, c) N 1*s*, d) O 1*s* and Sb 3*d*, e) Cu 2*p*, f) S 2*p*.

**Figure** **3**a presents the surface chemical composition observed as: C: 23.9%, N: 5.1%, O: 5.6%, S: 15.1%, Cu: 17.1%, and Sb: 33.1%. The high resolution C 1*s* core level spectrum of sample Cu_1_Sb_1.5_S/CF (Figure 3b) can be deconvoluted into three contributions at 284.8 eV (74%), 286.2 eV (19%), and 288.0 eV (7%), assigned to adventitious carbon/─C─C─/─C═C─, C─OH/─C═N, and ─COOH/─COO^−^, respectively. The contribution due to C═O, observed in the case of sample CF, is not observed. The N 1*s* spectrum shows a broad peak centered at 400.1 eV (Figure 3c assigned to ─C═N. The O 1*s* signal cannot be analyzed because it is fully overlapped with the Sb 3*d*_5/2_ signal. For this reason, we used the Sb 3*d*_3/2_ (Figure 3d) that appears at 539.5 eV, typical of Sb(III). This confirms the co‐existence of two chemical states of Sb^5+^ in Cu_3_SbS_4_ and Sb^3+^ in Sb_2_S_3._ The high‐resolution Cu 2*p* core level spectrum(Figure 3e) presents a Cu 2*p*_3/2_ at 932.0 eV without satellite. This binding energy value is typical of copper sulfide. Finally, Figure 3f shows the 2*p* core level spectrum presents two doublets S 2*p*_3/2_‐S 2*p*_1/2_ with S 2*p*_3/2_ binding energy values at 161.8 and 168.3 eV, assigned to sulfide and sulfate, respectively, being the former the predominant. These findings demonstrate that the hydrothermal growth of Sb_2_S_3_ and Cu_3_SbS_4_‐Sb_2_S_3_ on CF does not affect the elemental states/electronic structure of CF.

**Figure 4:**
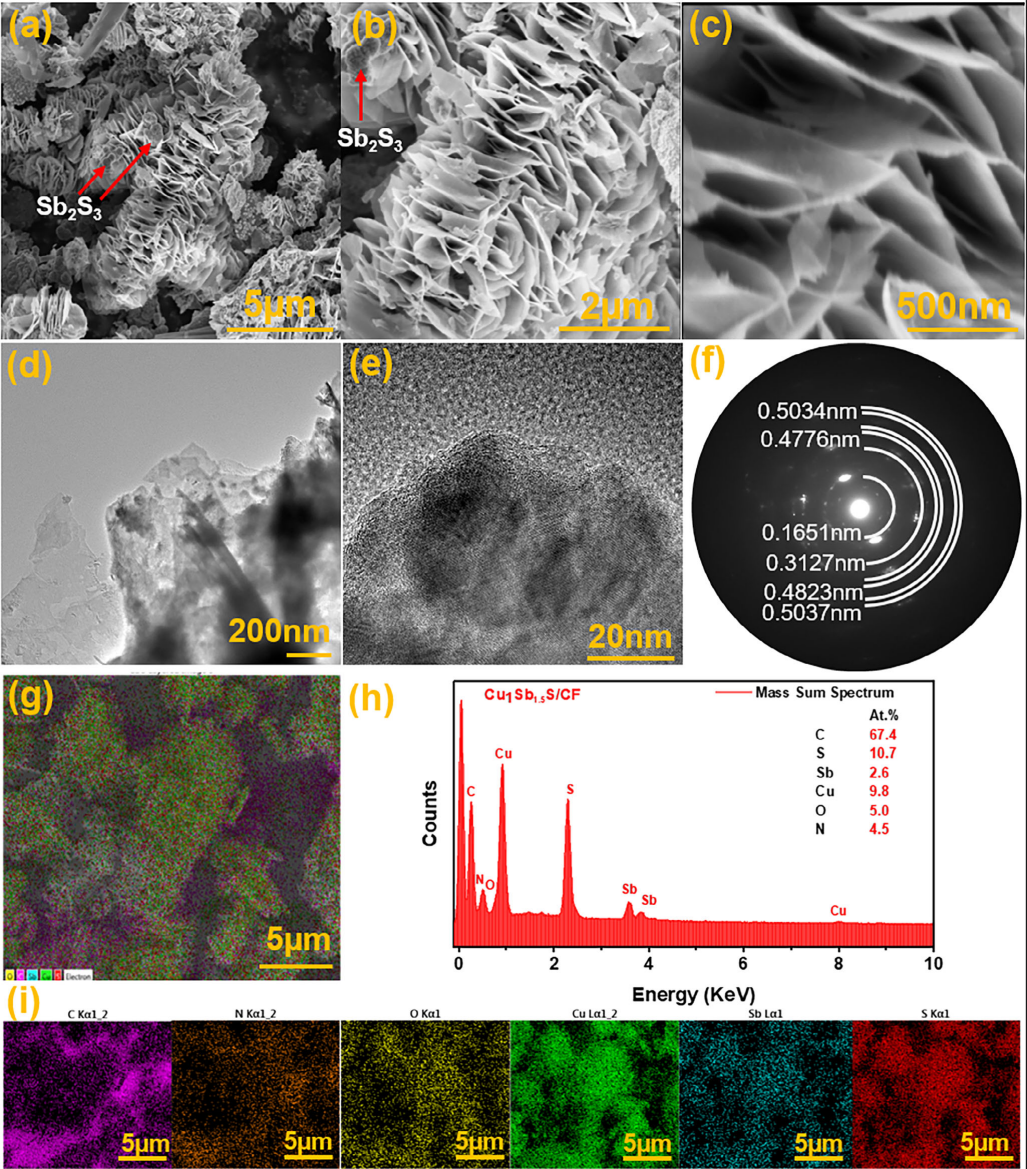
a–c) SEM image of Cu_1_Sb_1.5_S/CF, d–f) HR‐TEM of Cu_1_Sb_1.5_S/CF, g–i) elemental mapping and EDS mass sum spectrum of Cu_1_Sb_1.5_S/CF.

Scanning electron microscopy (SEM) analyses was performed to investigate the surface morphology of bare CF, SbS/CF, and Cu_1_Sb_1.5_S/CF. In Figure S4a,b (Supporting Information), the as‐synthesized CF surface presents the open cell pores developed due to the physical templating effect of molten salt and the foaming feature occurred as a result of carbon dioxide and water molecules released during degradation of PET.^[^ ^15^ ^]^ In literature, some recent studies present a similar morphology of carbon foam with tiny open pores and flat sheets of CF, indicating its graphitic nature.^[^ ^35^ ^]^ The surface morphology of the stibnite phase of Sb_2_S_3_ on CF shows the elongated, densely packed nanorods that cover the CF surface, as shown in Figure S5a,b (Supporting Information). The nanorods present a good resemblance to literature‐reported hydrothermally grown nanorods of stibnite Sb_2_S_3_.^[^ ^36^ ^]^ Upon introducing Cu into the reaction system (representing the heterostructure of Cu_3_SbS_4_‐Sb_2_S_3_ on CF), a significant morphological transformation is observed. The nanorods evolve into vertically aligned nanosheets as shown in **Figure** **4**a–c with different magnifications. Interestingly, it exhibits interconnected layers, forming abundant open channels that can facilitate mass transfer which is an advantageous feature for interfacial solar steam generation. Surface morphology plays a crucial role in the efficient performance of solar evaporators, and the aligned nanochannels in nanostructures facilitate directional water transport by reducing the vapor escape resistance and heat localization at the evaporation surface due to enhanced light absorption. The elemental compositions of the samples were investigated through energy‐dispersive X‐ray spectroscopy (EDS), and the results are presented in Figure S4 (Supporting Information), Figure 4, and Figure S5 (Supporting Information), respectively. The elemental mapping shows the uniform distribution of elements in each respective sample. The TEM image in Figure 4d further confirms the nanosheet morphology seen in SEM. The thin, wrinkled edges in the TEM image indicate the presence of ultrathin sheet‐like domains, consistent with the SEM observation. The contrast variations suggest a heterogeneous structure, possibly due to the coexistence of Cu_3_SbS_4_ and Sb_2_S_3_ phases within the nanosheets. The existence of the two phases is further corroborated by the HRTEM analysis (Figure 4e), which reveals the distinct lattice fringes that are consistent with the crystal planes of Cu_3_SbS_4_ and Sb_2_S_3_. The obtained d‐spacing (SAED) values of 0.48, 0.32, and 0.16nm, were successfully matched to the corresponding XRD crystal planes of Cu_3_SbS_4_ (101), (112), and (312); and the d‐spacing 0.503nm corresponds to Sb_2_S_3_ crystal planes (020), (120). These observations confirmed the successful synthesis of the heterostructure with retained crystallinity.

**Figure 5:**
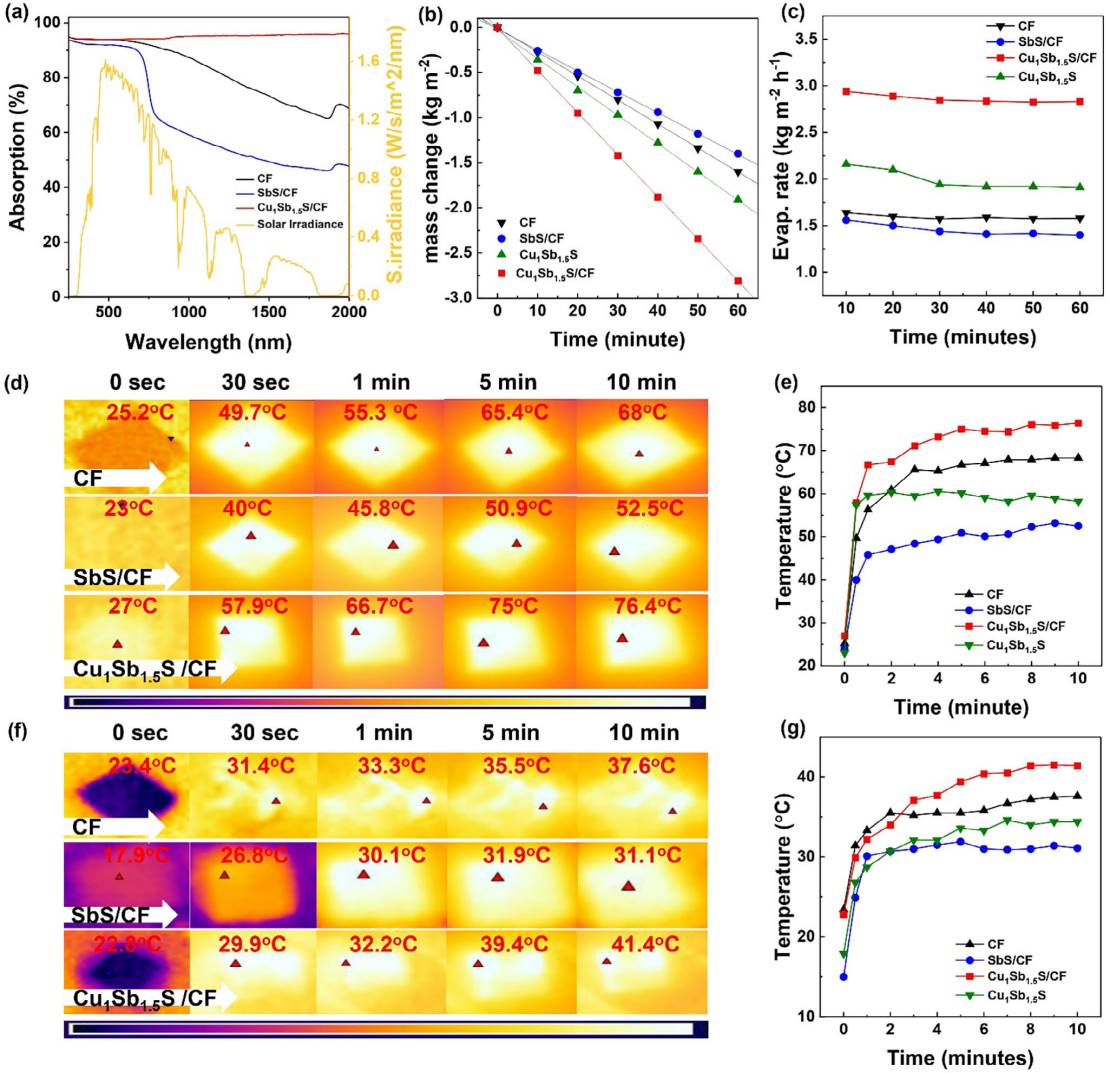
a) UV–vis absorption analyses, b,c) Change in mass and evaporation rate of solar desalination, d,e) thermal images and graph of dry surface temperature of CF, SbS/CF, Cu_1_Sb_1.5_S, and Cu_1_Sb_1.5_S/CF, f,g) thermal images and graph of wet surface temperature of CF, SbS/CF, Cu_1_Sb_1.5_S, and Cu_1_Sb_1.5_S/CF.

Efficient photothermal conversion relies critically on the ability of materials to absorb a broad spectrum of solar radiation, particularly spanning the ultraviolet (UV), visible (Vis), and near‐infrared (NIR) regions, where the majority of solar energy is concentrated.^[^ ^37^, ^38^ ^]^ To understand the fundamental optical properties, the absorption spectra of the synthesized samples were recorded across the UV/Vis to Near‐IR range. Figure S6 (Supporting Information) shows the results of bare CF, SbS, and SbS/CF, and various composites with different Cu/Sb ratio (1:1, 0.5:1, 1.5:1, 1:0.5, and 1:1.5). The selected sample demonstrate optimum ratio Cu_1_Sb_1.5_S/CF (i.e equivalent to Cu_3_SbS_4_‐Sb_2_S_3_/CF) that delivers the highest absorption is also depicted in **Figure** **5**a. The sample Cu_1_Sb_1.5_S/CF exhibits an almost constant absorption ranging between 94% and 96% in the full 300‐2000 nm spectral range. Bare CF and SbS/CF samples exhibit a significant drop in absorbance in the NIR region. Specifically, the Sb_2_S_3_ functionalized CF shows a dramatic decrease in absorption from 90% in UV/Vis region down to 48% (at 2000 nm) in the near‐IR region, most likely due to the wide band gap of stibnite Sb_2_S_3_. Although Sb_2_S_3_ presents excellent absorption in UV/Vis region, but declined in absorption activity in NIR is because of higher wavelength (>800nm) and the photon with lower energy does not absorb well due to integration of Sb_2_S_3_ with CF, the Sb_2_S_3_ layer partially masks the highly NIR‐absorptive carbon surface with a material that performs poorly in NIR region. The uniform coating of Sb_2_S_3_ nanocrystals can increase the surface reflectance of the material that interferes with the CF. Both factors, the spectral mismatch and structural interference, explain why SbS/CF shows lower overall light absorption than bare CF. The enhanced absorption of Cu_1_Sb_1.5_S/CF is achieved due to the heterojunction of famatnite (Cu_3_SbS_4_) and stibnite (Sb_2_S_3_: Such a heterojunction of Cu_3_SbS_4_ with Sb_2_S_3_ extends the high absorption in the near‐IR region. For instance, the study on optical activity for famatnite (Cu_3_SbS_4_) has been reported, illustrating the high optical absorption in near‐IR due to its narrow bandgap of 0.9 eV.^[^ ^39^ ^]^ Another study reported the excellent optical absorption of Cu_3_SbS_4_ nanocrystals in the UV/Vis‐NIR region,^[^ ^40^ ^]^ which demonstrates its potential as a photothermal material. Based on the comprehensive material structural characterization, especially optical absorption, the synthesized heterostructure could be an excellent material for the interfacial solar desalination process.

The interfacial solar evaporation experiment was performed under simulated solar light (AM 1.5G one sun irradiation 100 mW cm^−2^), the experimental detailed setup is illustrated in Figure S7a (Supporting Information). For a detailed comparison, the solar desalination test was conducted on different samples:CF, Cu_1_Sb_1.5_S/CF, SbS/CF, and Cu_1_Sb_1.5_S without CF. Figure 5b presents the mass change of the investigated samples, which elucidates the continuous reduction of water mass. The obtained results in Figure 5c reveal the excellent evaporation rate of 2.83 kg m^−2^ h^−1^ for Cu_1_Sb_1.5_S/CF compared to the bare CF (1.58 kg m^−2^ h^−1^) and SbS/CF (1.4 kg m^−2^ h^−1^). The outperforming evaporation rate of Cu_1_Sb_1.5_S/CF is attributed to the synergistic photothermal effect of Cu_3_SbS_4_ and Sb_2_S_3_. The improved results achieved due to the complementary absorption behavior of Cu_1_Sb_1.5_S/C and the integration of Cu_3_SbS_4_ enable a broader spectral response compared to Sb_2_S_3_ functionalized CF, and their combined effect facilitates interfacial charge separation under solar irradiation within a heterostructure that boosts the energy to be dissipated non‐radiatively as heat. The alignment of absorption activity (UV/Vis‐NIR) with surface temperature (Cu_3_SbS_4_–Sb_2_S_3_/CF > CF > Sb_2_S_3_/CF) indicates the heterostructure facilitates more effective solar‐to‐thermal energy conversion. Figure 5d,e presents the thermal images and graphs of the investigated samples that were captured at the dry state (before desalination), Figure 5f,g presents the wet condition (during desalination). At the dry state Figure 5d,e, the Cu_1_Sb_1.5_S/CF composite exhibits the highest surface temperature, reaching 76.4 °C, significantly surpassing that of pristine CF (68.3 °C) and SbS/CF (52.4 °C). Figure 5f,g shows the operational photothermal performance of the materials under wet conditions, which presents the relative trend consistent with the dry state of high photo‐to‐thermal conversion. Accordingly, the Cu_1_Sb_1.5_S/CF composite shows the highest wet surface temperature of 41.4 °C followed by CF (37.6 °C) and SbS/CF (31.1 °C). During operational conditions at the wet surface, the lowering of temperature is obvious due to the cooling effect of saline water. The superior photo‐to‐thermal conversion indicates the effective radiation absorption (according to the optical absorption results) that maintained the heat localization at the wet‐air interface. This thermal behavior under working conditions relates well to its highest evaporation rate. As revealed by scanning electron microscopy (SEM) of the investigated samples, it provides further insights to understand the crucial role of the surface morphology of solar evaporators. The vertically stacked nanosheets (nanochannels) increase internal multiple light reflections that help to trap solar photons. In results, it concentrates the photothermal energy at the interface that retains the produced heat locally, decreasing the thermal distribution into the bulk or surrounding water. The effect is proven with infrared imaging demonstrating explaining higher surface temperatures for the heterostructure. In comparison SbS/CF) rod‐like morphology presents less surface interface for light interaction, fewer scattering due to fewer trapped photons, resulting in poorer photothermal and evaporation performance. The formation of the composite not only provides high absorption from UV/Vis‐NIR but also facilitates the water transport due to nanochannels formation. The surface morphology plays a pivotal role in enhancing the performance of photothermal evaporators. Another interesting point is the comparison we made between Cu_1_Sb_1.5_S (1.9 kg m^−2^ h^−1^) *vs* Cu_1_Sb_1.5_S/CF (2.83 kg m^−2^ h^−1^). These findings indicate the significant contribution of the CF in addition to the optimum heterojunction between Cu_3_SbS_4_ and Sb_2_S_3_. In fact, the unique hydrophilic pores in the CF architecture provide high water transport toward the photothermal layer. In recent findings, Bai et al demonstrate similar outcomes for carbon foam/PVDF that show the evaporation rate of 1.27 kg m^−2^ h^−1^ due to its hydrophilicity.^[^ ^15^ ^]^ Furthermore, the present study shows much improved results in comparison to our recently reported work on CuS functionalized CF showing an evaporation rate of 1.92 kg m^−2^ h^−1^.^[^ ^41^ ^]^

**Figure 6:**
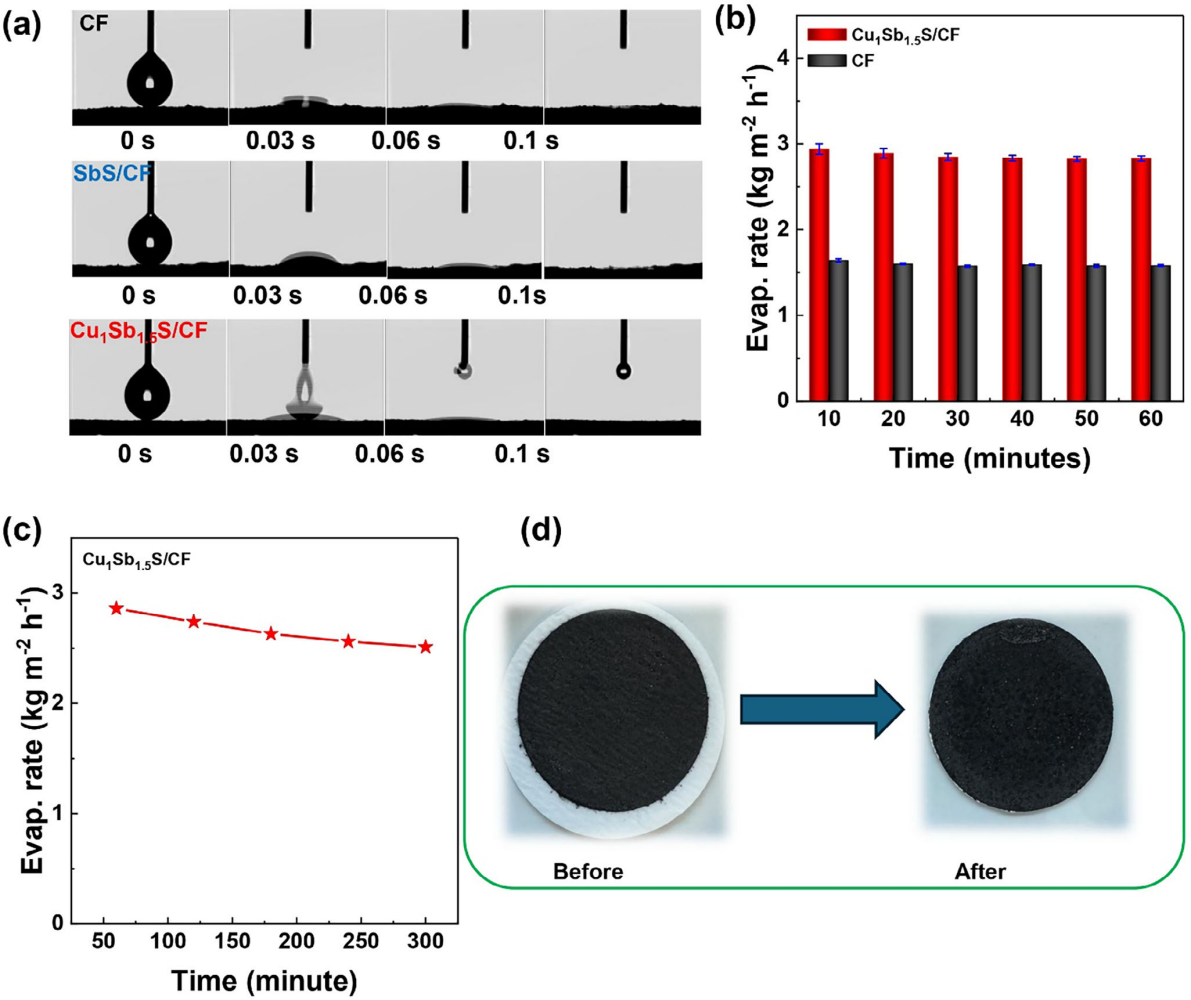
a) contact angle analysis, b) evaporation rate with error bar c) stability graph of Cu_1_Sb_1.5_S/CF on 3.5% NaCl saline water under one sun irradiation, and d) Real photographs of Cu_1_Sb_1.5_S/CF before and after stability test.

To provide experimental evidence on the hydrophilicity, the surface wettability using contact angle analysis was performed for the pristine CF and Cu_1_Sb_1.5_S/CF. **Figure** **6**a shows the instantaneous water droplet absorption in less than a second for both samples. This evidence highlights the hydrophilic character of CF, which is attributed to the presence of oxygen and nitrogen in the graphitic skeleton of CF.^[^ ^15^, ^41^ ^]^ Hydrophilicity is a crucial factor in solar desalination to achieve a high evaporation rate, in the waste PET‐derived carbon foam, the oxygen and nitrogen containing surface functional groups introduced during the carbonization of PET impart high wettability and surface energy to the CF, which allows water to be rapidly drawn into and foster the capillary action of vapor evaporation. As seen in the surface morphology of carbon foam in Figure S4a (Supporting Information) it presents tens of open‐cell pores architecture formed due to the physical template effect of molten salts and foamy effect of in situ formed tiny molecular compounds such as CO_2_ and H_2_O in the degradation and crosslinking process in the CF; these structural features act as continuous capillary channels for ensuring consistent water availability at the evaporation interface. The contact angle observation further solidifies these results, with rapid absorption of water droplets confirming the high wettability that drives the capillary movement of water from the bulk reservoir upward to the photothermal surface. Hu et al have demonstrated in a study on waste‐derived carbon foam that wettability is a function of oxygen‐containing groups and a proportional relation of the number of pores on the surface, which results in capillary movement of water molecules.^[^ ^13^ ^]^ The Cu_1_Sb_1.5_S/CF nanocrystal decoration does not alter CF hydrophilic characters, is a requisite for efficient water transport and high evaporation at the interface due to constant water supply. The interfacial desalination process for the bare sample CF and the outperforming solar evaporator Cu_1_Sb_1.5_S/CF was conducted thrice, the evaporation rate was measured every 10 min, and the mean evaporation rate is presented with the error bar in Figure 6b. The evaporation efficiency of Cu_1_Sb_1.5_S/CF is calculated from the value obtained for water vaporization enthalpy from DSC (Differential Scanning Calorimetry) analysis. The Cu_1_Sb_1.5_S/CF sample presents an excellent evaporation efficiency of 94.4%, supporting the performance of the synthesized solar evaporator. The evaporation efficiency is a key parameter (as well as the evaporation rate) to evaluate the potential of solar evaporators for desalination.^[^ ^42^ ^]^ The present results outperform to recently reported carbon‐derived solar evaporators, such as: MOF‐porous carbon 64.41%, Co‐N─C/CF 87%, and Carbon nanoparticle fabric 76%.^[^ ^43^, ^44^, ^45^ ^]^

The evaporation rate is a key parameter when evaluating the potential of solar evaporators. The stability test in this context ensures that this rate remains consistent, predictable over time, and reliable. Due to a high saline aqueous environment, reporting of the evaporation rate over a longer experimental time demonstrates how gradually salt accumulation affects the evaporation rate. The stability test was performed under one sun irradiation using a glass microfiber filter (Whatman GF/F, CAT No. 1825‐047). The stability test presents a decline in evaporation from 2.86 to 2.51 kg m^−2^ h^−1^ in five hours Figure 6c. To confirm the salt crystallization on the surface of the solar evaporator, the real photographs were recorded before and after desalination. Figure 6d illustrates that there is no significant visual appearance of salt crystals on the surface, and the observed minor decline (∼12.24%) in evaporation rate might have occurred due to saturation of salt concentration in the remaining water or in the cotton sponge that works as a water transport toward photothermal evaporators. The minor decline over the test interval signifies good operational stability of the photothermal system. Post‐stability characterization was performed to assess the structural integrity and operational durability of the Cu_1_Sb_1.5_S/CF solar evaporator after five hours of prolonged use. As shown in Figure S8 (Supporting Information), the XRD patterns remain essentially unchanged, with no detectable shifts in peak positions or emergence of new phases. This clearly confirms that the crystalline structure of Cu_1_Sb_1.5_S/CF remains stable under illumination and extended solar exposure. The Cu_1_Sb_1.5_S/CF heterostructure exhibits exceptional desalination performance. For instance, the sodium concentration before the desalination experiment (the saline feed water) was 1.2838 × 10⁴ mg/L^−1^. Following the solar‐driven evaporation experiment, we have condensed back the evaporated water and analyzed the sodium content by inductively coupled plasma optical emission spectroscopy (ICP‐OES), which shows a significantly reduced sodium concentration of only 1.5 × 10^1^ mg/L^−1^. This corresponds to a remarkable salt removal efficiency of ≈99% (Figure S7c, Supporting Information). To further solidify this finding, the ohmic resistance of condensed water and the feed saline water (before and after desalination) was also recorded. For both sample analyses, an equal amount of sample volume (5 mL) was taken in a beaker with an internal diameter of 3 cm, the ohmic value was recorded using a Digital Multimeter (DM01M), and the distance between the meter electrode was ∼1.5 cm. Figure S7c (Supporting Information) shows that the ohmic resistance of the condensed purified sample (5.02 MΩ) is significantly higher compared to saline water (0.327 MΩ). These observations indicated the dramatic decrease in salt concentration in purified water that ultimately reduced the conductivity of the water sample.

**Table** **1** presents a detailed comparison of the evaporation rate of the recently reported solar steam generators with the present finding. Our work confirms the critical role of composite engineering for photothermal‐derived desalination, the enhanced performance of Cu_1_Sb_1.5_S/CF achieved due to the combined effect of CF presenting high hydrophilicity with low thermal conductivity (0.113 W m^−1^ K^−1^)^[^ ^37^ ^]^ and photothermal effect of Cu_3_SbS_4_ and Sb_2_S_3_ in enhancing both photothermal conversion and water evaporation performance, emphasizing the potential of composite microstructures for efficient solar desalination applications.

**Table 1:** Comparison of Cu_1_Sb_1.5_S/CF with recently reported carbon‐based nano solar steam generators under one sun irradiation.

Solar Evaporators	Evap. Rate [kg m^−2^ h^−1^]	Refs.
N‐doped carbon foam	1.39	[46]
MoS_2_‐loaded carbon foam	1.48	[47]
3D Janus foam	1.78	^[^ ^48^ ^]^
Wood slice loaded Pd, Au, Ag nanoparticles	1.15	[49]
carbon‐coated Fe_3_O_4_ NPs	1.07	[50]
CuS/polyethylene membrane	1.02	[51]
GO/ Ti_3_C_2_T_x_ MXene	2.09	[52]
Co‐N‐C/CF	1.88	[44]
CuS‐carbon foam	1.92	[41]
NiSe‐hyd	1.85	[53]
CoSb_x_	1.40	[54]
HEAO/PDMS‐coated wood	1.90	[55]
Cu_3_SbS_4_‐Sb_2_S_3_/CF	2.82	Present study

## 3 Conclusion 

Interfacial solar desalination is an emerging technology for the production of fresh water utilizing renewable solar energy. The present study illustrates the upcycling of waste PET plastic into CF and further hydrothermal decoration of CF through Cu_3_SbS_4_‐Sb_2_S_3_ to prepare a solar steam generator. XRD and XPS analysis confirm the crystal structure and chemical state of the synthesized materials, and SEM‐EDS present the nanochannels morphology. Optical analysis (UV/Vis‐NIR) indicates excellent optical absorption (96%) that provokes the high photothermal output. Bridging the advantage of morphological transformation and the synergistic effect of heterostructuring, an enhanced evaporation rate of 2.82 kg m^−2^ h^−1^ was obtained, with 99% removal of salt concentration. These findings suggested the sustainable development of engineered nano‐evaporators from chalcogenide‐functionalized waste plastic‐derived carbon material, which represents the rational design of a solar steam generator to enable circular economy practices.

## 4 Experimental Section

All the chemicals and reagents used in the present research work were purchased from Sigma Aldrich: CuCl_2_.2H_2_O, SbCl_3_.5H_2_O, NaCl, ZnCl_2_, Melamine, HCl, and thiourea.

### Synthesis of Cu_3_SbS_4_‐Sb_2_S_3_/CF material

Initially, the carbon foam was synthesized by using the procedure reported in,^[^ ^15^ ^]^ In detail, 1.5g of waste PET bottle sheet was mixed with 0.75g of melamine and 3g of eutactic salt (ZnCl_2_/NaCl (58/42)) and carbonized at 340 °C for 30 min with a heating rate of 10 °C/min. After cooling to room temperature, the obtained product was washed with 0.5M HCl and distilled water repeatedly until neutral pH, and then dried at 60 °C (labelled as CF). The functionalization of CF with Cu_3_SbS_4_‐Sb_2_S_3_ was carried out using a hydrothermal synthesis approach. 1mM CuCl_2_.2H_2_O and 1.5mM SbCl_3_.5H_2_O were mixed in 100 mL of distilled water and sonicated for 20 min, the mixture was placed on a magnetic stirrer with the addition of 0.15g of CF and 2mm of thiourea and further stirred for 30 min. After stirring, the precursor mixture was transferred into a Teflon‐lined stainless autoclave and placed in an oven for 24 h at 180 °C. After cooling to room temperature, the black powder was centrifuged with several washes of distilled water and ethanol. Finally, the obtained materials were dried at 60 °C in a drying oven and placed in a desiccator. The same procedure was followed to synthesize material with different ratios of Cu and Sb (1:1, 1.5:1, 0.5:1, 1:1.5 and 1:0.5) in order to optimize the best combination and labeled as Cu_1_Sb_1_S/CF, Cu_1.5_Sb_1_S/CF, Cu_0.5_Sb_1_S/CF, Cu_1_Sb_1.5_S/CF, and Cu_1_Sb_0.5_S/CF. The same reaction time, temperature, and CF concentration were followed to synthesize Sb_2_S_3_ on CF with 1mM SbCl_3_.5H_2_O and 2mM thiourea, and the final product was labeled as SbS/CF.

### Characterization

The UV/Vis absorption spectroscopy analysis (UV/Vis‐NIR) was performed using an ultraviolet‐visible spectrophotometer LAMBDA 1050^+^, Perkin Elmer with an integrating sphere. A Philips PW1050/37 diffractometer was used to carry out the X‐ray diffraction (XRD) analysis. X‐ray photoelectron spectroscopy (XPS) was performed using an ESCALAB 250 (Thermo Fisher Scientific USA) spectrometer with an x‐ray source monochromated Al Kα 150W and energy pass 200 eV for the survey and 30 eV for high‐resolution scans. The surface morphology and elemental mapping (SEM‐EDS) analysis were carried out using an FEI Magellan 400 FEG‐SEM with EDS detector, X‐Max 80 mm^2^ SDD (Oxford Instrument). The HR‐TEM (High‐resolution Transmission Electron Microscopy) micrographs were obtained via the JEOL ARM 200F microscope with 2 CS correctors of CC3M competence center of Institut Jean Lamour. The microscope was equipped with an MSC794 camera Gatan one view, JEOL HAADF detector, and a double‐tilt sample holder. Hydrophilicity analysis was performed using the FTA 1000 Analyzer System for the contact angle measurements. Differential Scanning Calorimetry (DSC) analysis was carried out using a 1020 Series DSC 7 thermal analysis system, with the following temperature range 20 °C to 150 °C at 5 °C/min. The Inductively Coupled Plasma Optical Emission Spectroscopy (ICP‐OES) was performed with a Perkin Elmer Optima 5300SV spectometer.

### Solar Desalination

The interfacial solar desalination experiments were conducted by preparing photothermal evaporators through vacuum filtration. Specifically, 0.1 g of the material was first dispersed in 20 mL of distilled water and sonicated to ensure uniform suspension. The dispersion was then deposited onto a glass microfiber membrane (Whatman GF/F, CAT No. 1825‐047) using vacuum filtration. After filtration, the membrane was dried at 60 °C to enhance film adhesion and mechanical stability. The resulting coated membrane served as the active photothermal layer in the solar evaporator. Then, 40 mL brine solution was taken in a glass beaker (0.6 m NaCL solution in the deionized water that was 3.5 wt.% average global seawater salinity) covered with polyethylene foam as a thermal insulator. A piece of membrane, 1x1 cm^2,^ cut, was placed on the cotton sponge that facilitates water transport to the membrane and passes through the thermal insulator. The solar desalination experiment was performed under one sun radiation (air mass 1.5 global, AM1.5G, 100 Wcm^−2^), and the relative humidity was 35%. The surface temperature of the membrane was recorded in the dry state and during the desalination process using a thermal camera (FLIR C3‐X). The evaporation rate of the material was calculated using the following equation;

(1) $$Evaporationrate=\frac{\Delta m}{S}\times \tau \;\;$$

where Δm is the mass loss of water (kg), S is the area of the evaporation (m^2^), and τ is the evaporation time (h).

Evaporation efficiency (𝜂) is calculated by using the following equation;

(2) $$\eta =\frac{mh_{lv}}{C_{opt}q_{i}}$$

where m is the mass flux kg m^−2^ s^−1^, h_lv_ is the enthalpy of liquid, C_opt_ is the optimal concentration, and q_i_ is solar radiation 1KW/m^2^.

## Conflict of interest

The authors declare no conflict of interest.

## Supporting information



Supporting Information

## Data Availability

The data that support the findings of this study are available from the corresponding author upon reasonable request.
