# Supplementary material for: Upcycled PET‐Derived Carbon Foam Functionalized with Cu3SbS4–Sb2S3 Heterostructures for Efficient Interfacial Solar Desalination
Source: Small. 2025 Aug 20;21(39):e06862. doi: 10.1002/smll.202506862 (PMC12490173; doi:10.1002/smll.202506862)
Supplement: Supplementary file 1 — Supporting Information [file SMLL-21-e06862-s001.docx]

**Upcycled PET-Derived Carbon Foam Functionalized with Cu₃SbS₄–Sb₂S₃ Heterostructures for Efficient Interfacial Solar Desalination**

*Muzammil Hussain^a,b^, Kassa Belay Ibrahim^a^, Enrique Rodríguez-Castellón^c^, Silvia Gross^d^, Pawan Kumar^e^, Stéphanie Bruyère^f^, David Horwat^f^, Elisa Moretti^a^*, Alberto Vomiero^a,e*^, Tofik Ahmed Shifa^a*^*

^a^Department of Molecular Sciences and Nanosystems, Ca' Foscari University of Venice, Via Torino 155, 30172 Venice, Italy

^b^Department of Industrial Engineering, University of Padova, Via Venezia, 1, 35131 Padova, Italy

^c^Department of Inorganic Chemistry, Crystallography and Mineralogy, Faculty of Science, Inter-university Institute of Research in Biorefinery I3B, University of Malaga, Málaga, Spain

^d^Department of Chemical Sciences, University of Padova, Via Francesco Marzolo 1, 35131 Padova, Italy

^e^Division of Materials Science, Department of Engineering Sciences and Mathematics, Luleå University of Technology, 97187 Luleå, Sweden

^f^Université de Lorraine, CNRS, IJL, Nancy, F-54000 France

*Corresponding Authors:

Tofik Ahmed Shifa

Email: [tofikahmed.shifa@unive.it](mailto:tofikahmed.shifa@unive.it)

Alberto Vomiero

Email: [alberto.vomiero@ltu.se](mailto:alberto.vomiero@ltu.se)

Elisa Moretti

Email: [elisa.moretti@unive.it](mailto:elisa.moretti@unive.it)

**Supporting Information**

**
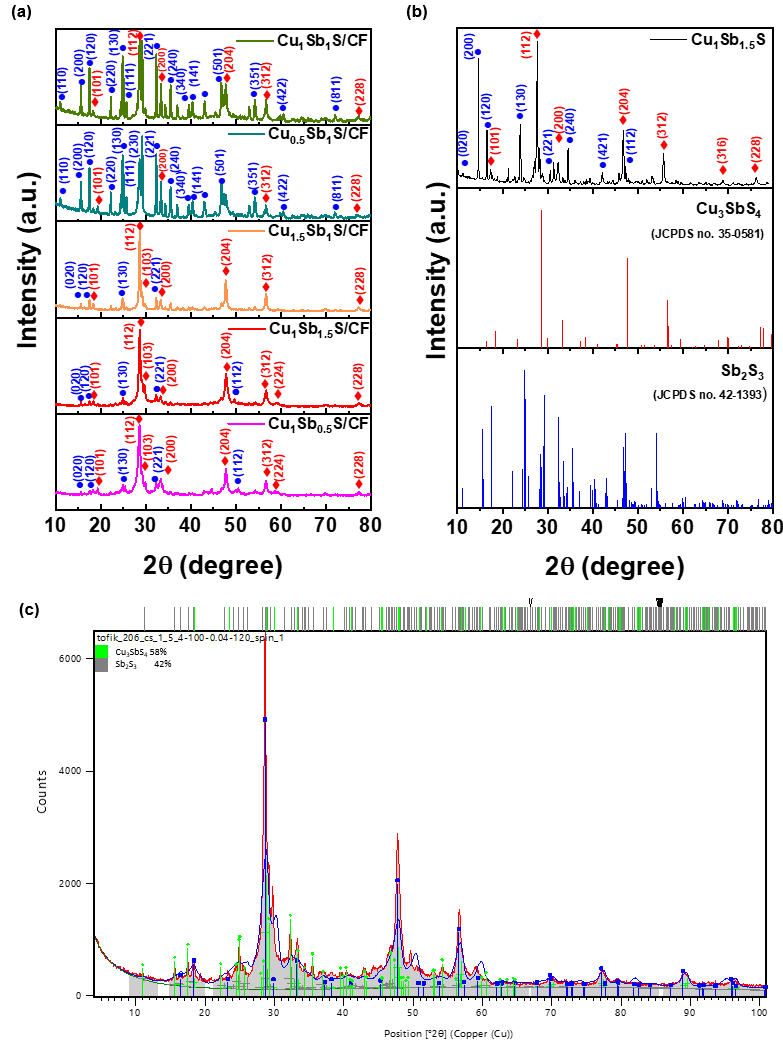
**

**Figure S-1.** (a) XRD pattern of the synthesized samples with varied concentration of Cu and Sb, (b) XRD pattern of Cu_1_Sb_1.5_S without CF, (c) XRD Rietveld Refinement pattern of Cu_1_Sb_1.5_S.


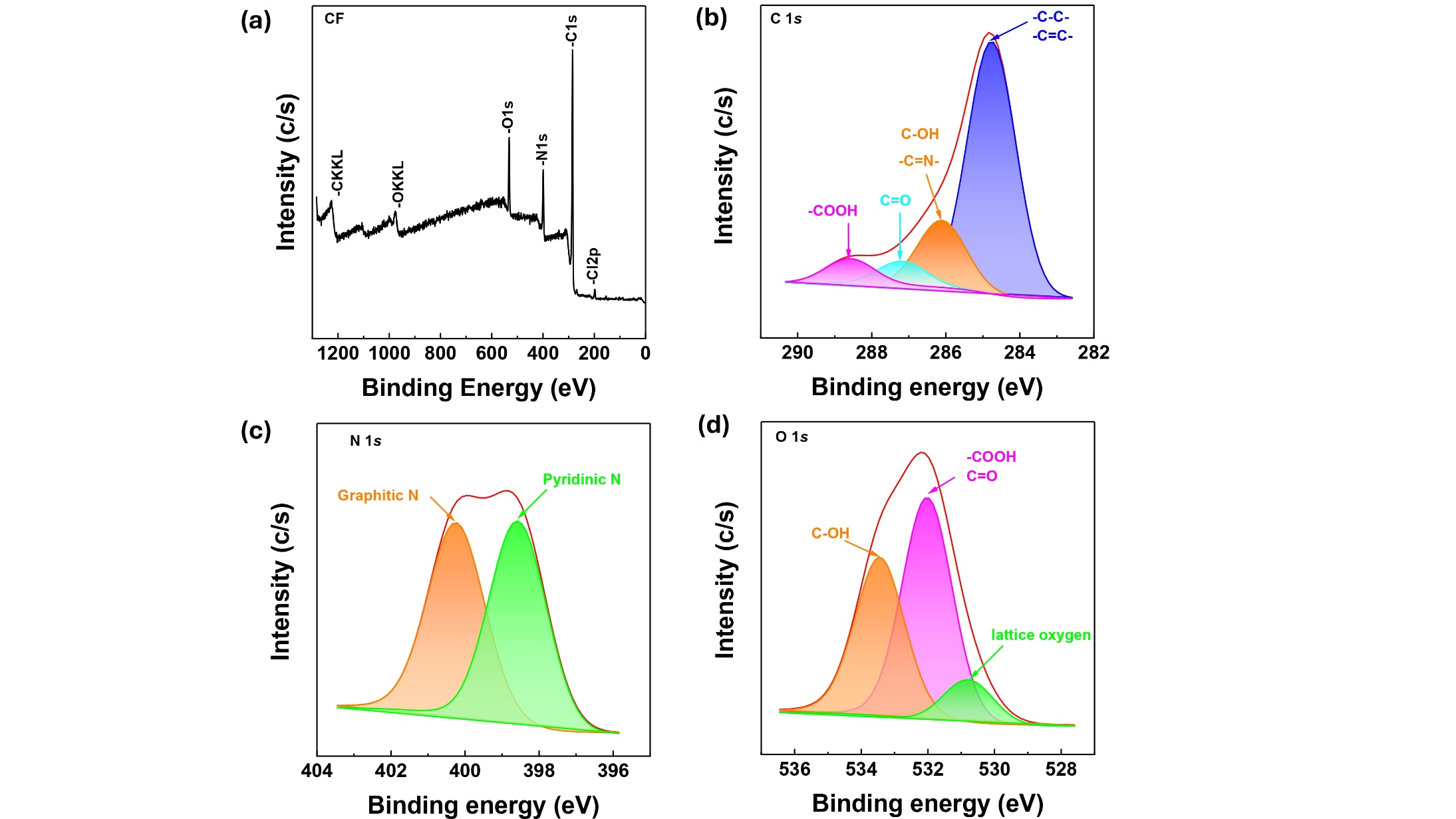


**Figure S-2.** (a) XPS survey spectrum of CF, high-resolution (b) C 1*s*, (c) N 1*s*, and (d) O 1*s* of CF.


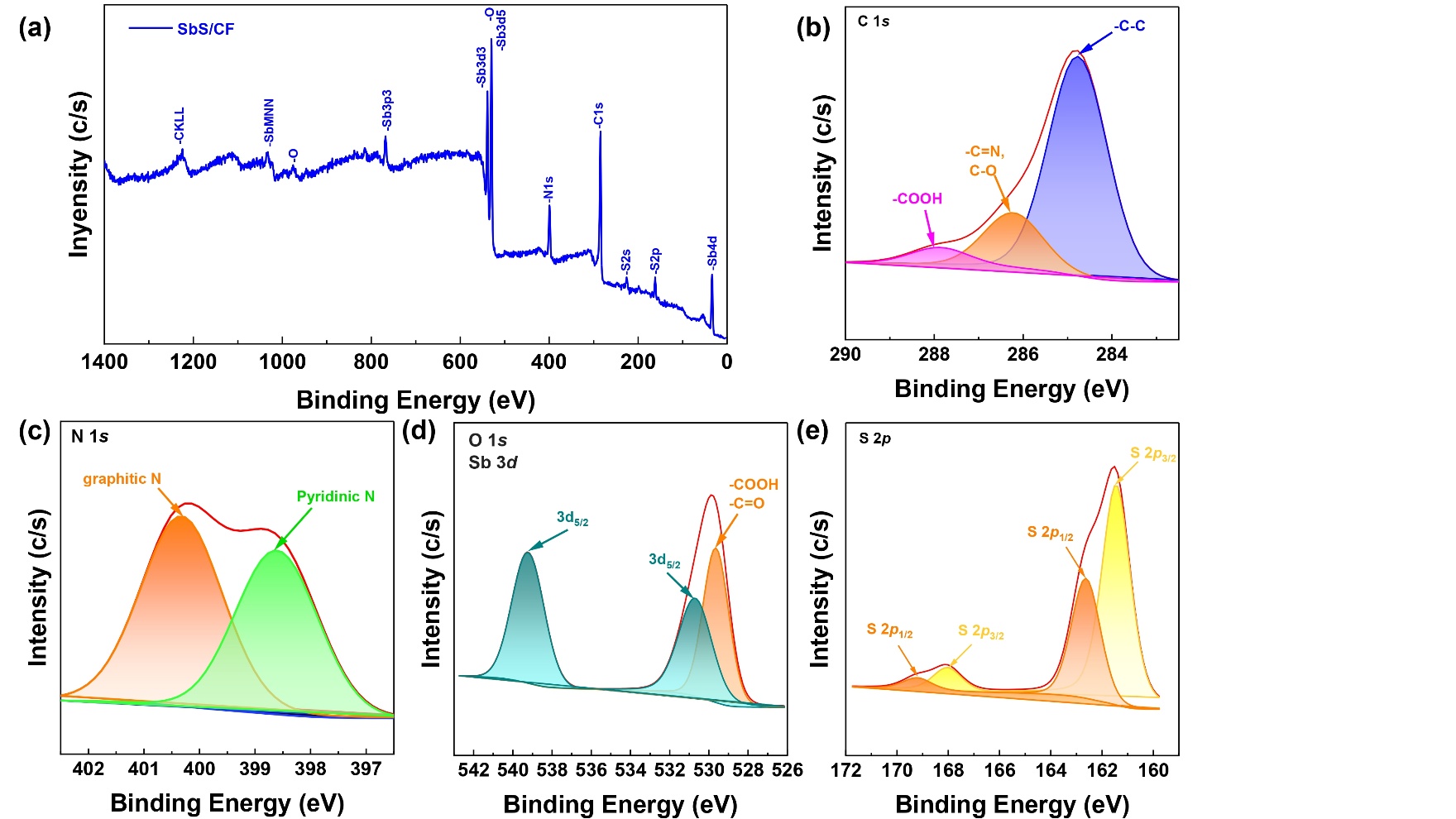


**Figure S-3.** (a) XPS survey spectrum of SbS/CF, high-resolution (b) C 1*s*, (c) N 1*s*, (d) O 1*s* and Sb 3*d*, and (e) S 2*p* of SbS/CF.


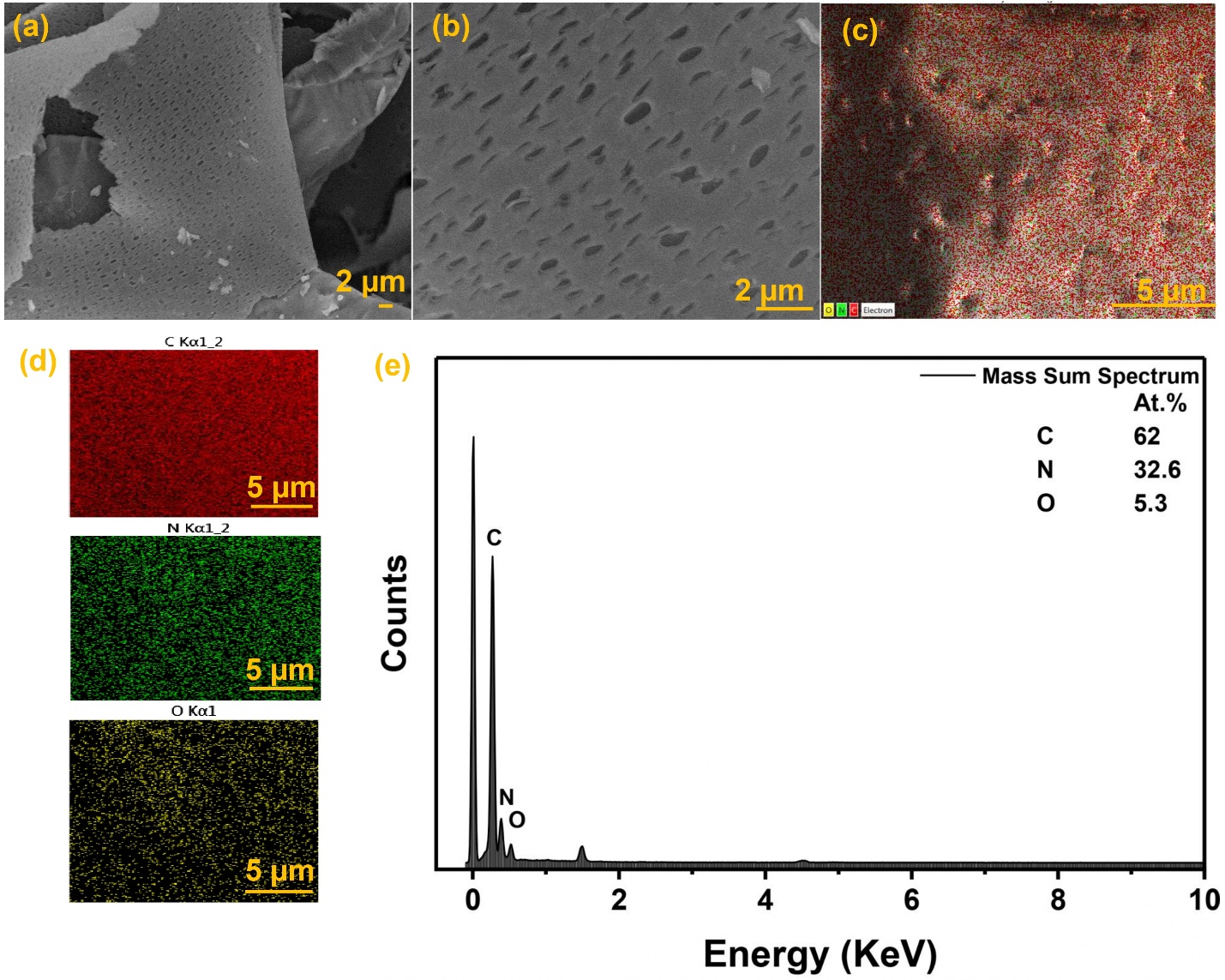


**Figure S-4.** (a-b) SEM images of CF, (c-e) elemental mapping and EDS spectra of CF.


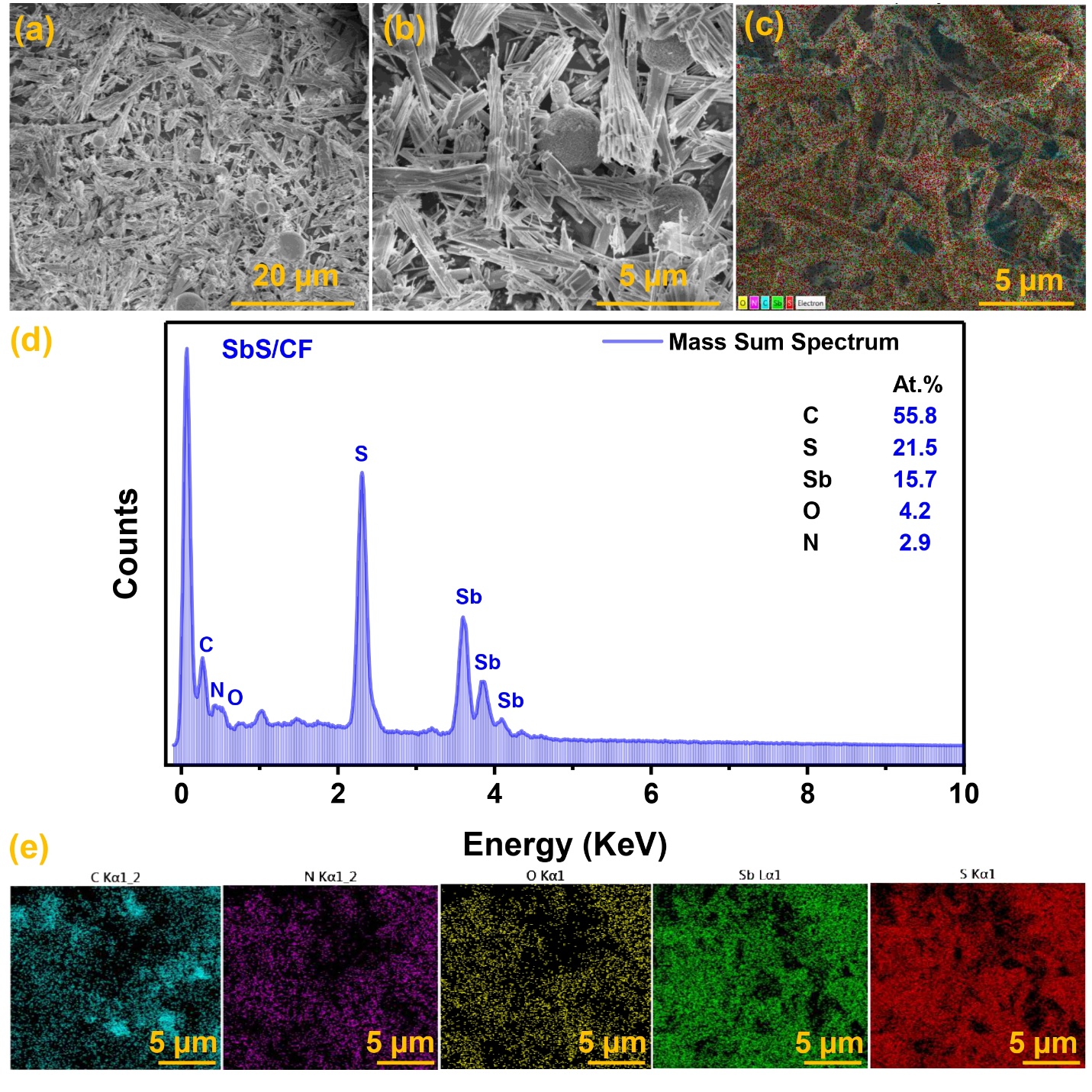


**Figure S-5.** (a-b) SEM images of SbS/CF, (c-e) EDS spectra, and elemental mapping of SbS/CF.

**Figure S-6.** UV/Vis-NIR spectra of all the synthesized samples.


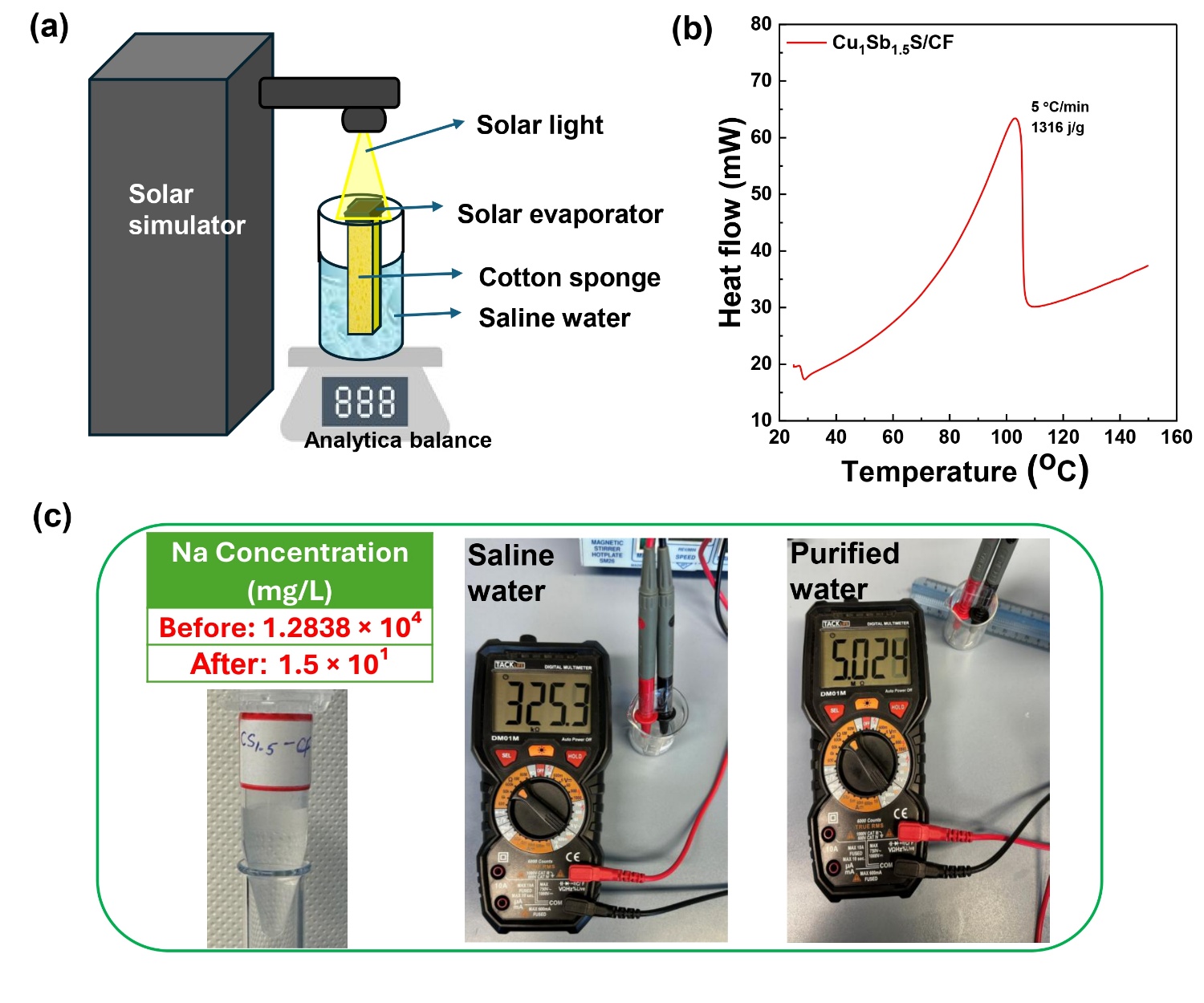


**Figure S-7**. (a) graphical illustration of solar desalination experiment, (b) DSC analysis, (c) Na concentration obtained from ICP-OES and resistivity measurement of saline and purified water.

**Figure S-8.** XRD pattern of Cu_1_Sb_1.5_S/CF before and after 300 minutes of solar desalination experiment.
